# Cell-Free Circulating Nucleic Acids as Early Biomarkers for NAFLD and NAFLD-Associated Disorders

**DOI:** 10.3389/fphys.2018.01256

**Published:** 2018-09-20

**Authors:** Andrey Turchinovich, Ancha Baranova, Oksana Drapkina, Alexander Tonevitsky

**Affiliations:** ^1^SciBerg e.Kfm, Mannheim, Germany; ^2^Molecular Epidemiology C080, German Cancer Research Center, Heidelberg, Germany; ^3^School of Systems Biology, George Mason University, Fairfax, VA, United States; ^4^Research Center for Medical Genetics, Moscow, Russia; ^5^Atlas Biomed Group, Moscow, Russia; ^6^Federal State Institution National Research Center for Preventive Medicine, Moscow, Russia; ^7^Department of Cell Biology, Higher School of Economics, Moscow, Russia; ^8^art photonics GmbH, Berlin, Germany; ^9^SRC Bioclinicum, Moscow, Russia

**Keywords:** cell-free RNA, NAFLD, early diagnosis, extracellular nucleic acids, circulating microRNA, liver diseases, liquid biopsy, non-coding RNA

## Abstract

Non-alcoholic fatty liver disease (NAFLD) is the worldwide most common cause of chronic liver pathology, which prevalence strongly correlates with the increasing incidence of diabetes, obesity and metabolic syndrome in the general population. Simple steatosis, the earliest NAFLD stage, usually remains asymptomatic, and appropriate changes in the lifestyle, as well as the diet, can reverse the affected liver into the healthy state. The potential of simple steatosis to progress into severe fibrotic stages and to facilitate carcinogenesis necessitates timely NAFLD detection and risk stratification in community-based healthcare settings. Since their initial discovery a decade ago, extracellular circulating miRNAs have been found in all human biological fluids including blood and shown to hold great promises as non-invasive biomarkers. Normally, intracellular miRNAs participate in the regulation of gene expression, but once released by dying/dead cells they remain highly stable in the extracellular environment for prolonged periods. Therefore, circulating miRNA profiles can reflect the ongoing pathogenic processes in body’s tissues and organs, and enable highly sensitive non-invasive diagnosis of multiple disorders. A non-urgent character of the NAFLD-related decision-making justifies the use of chronic liver diseases as an excellent test case for examining the practical utility of circulating miRNAs as biomarkers for longitudinal monitoring of human health. In this review, we summarize the state-of-the-art in the field of early diagnosis of NAFLD using circulating blood miRNAs, and stress the necessity of additional experimental validation of their diagnostic potential. We further emphasize on the potential diagnostics promises of other cell-free RNA species found in human biological fluids.

## Non-Alcoholic Fatty Liver Disease

**FIGURE 1:**
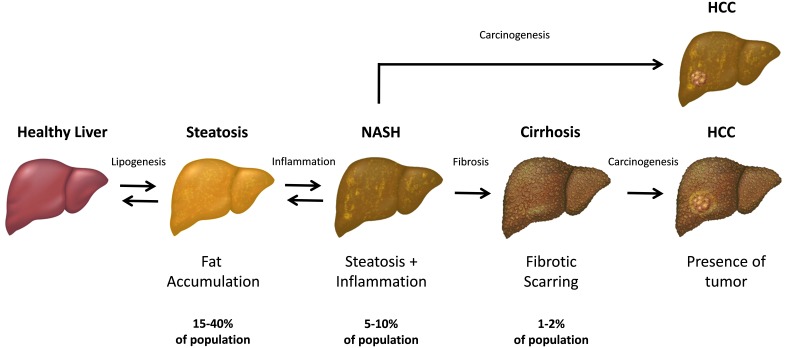
The progression and stages of non-alcoholic fatty liver disease (NAFLD). The steatosis (also referred as “simple steatosis” or “fatty liver”) is the initial NAFLD stage and is characterized by the excessive accumulation of fat in the hepatic cells. Upon developing inflammation, the liver steatosis may subsequently transform into NASH and finally, lead to the liver cirrhosis in some individuals. In turn, both NASH and cirrhotic liver has an increased chance to develop a hepatocellular carcinoma (HCC) within the affected parenchyma. Both simple steatosis and NASH are reversible stages of NAFLD, while the cirrhotic tissue cannot revert to the healthy state.

Non-alcoholic fatty liver disease (NAFLD) is a common chronic pathology associated with progressive histological alterations of the hepatic parenchyma (Marra et al., 2008; Starley et al., 2010; Chalasani et al., 2012). These NAFLD-associated changes range from a simple fat accumulation in hepatocytes, also known as hepatic steatosis or fatty liver, to a more severe histological picture, characterized by liver cell injury, fibrosis and inflammation, which hallmark the more advanced condition known as non-alcoholic steatohepatitis (NASH) (Marra et al., 2008; Starley et al., 2010) (**Figure 1**). Hepatic steatosis *per se* does not usually have any serious impact on health; however, in some individuals it may eventually progress into NASH, and subsequently, lead to more severe pathologies including liver cirrhosis and hepatocellular carcinoma (HCC) (Starley et al., 2010; Baffy et al., 2012; Michelotti et al., 2013). In fact, up to 20% of patients with NASH may develop cirrhosis (McCullough, 2004). Moreover, recent studies suggest that NAFLD may predispose patients to HCC even in the absence of cirrhosis through the linoleic acid-induced suppression of tumor surveillance (Ma et al., 2016). It is important to note that NAFLD is a multisystem disease that affects other organs including the pancreas, heart and cardiovascular system (Schwenger and Allard, 2014; Yki-Jarvinen, 2014). Consistent clinical and epidemiological data strongly indicate that simple steatosis is an independent risk factor for type 2 diabetes, coronary artery disease and cardiac mortality (Targher et al., 2007; Ratziu et al., 2010; Targher et al., 2010; Musso et al., 2011; Yki-Jarvinen, 2014). Finally, some research studies have shown that NAFLD-associated morbidity and mortality could be due to the cardiovascular complications rather than to the liver disease itself (Ekstedt et al., 2006; Rafiq et al., 2009; Torres and Harrison, 2015).

The prevalence of NAFLD has recently reached global epidemic proportions both in adults and children (Ratziu et al., 2010; Vernon et al., 2011; Welsh et al., 2013), representing a looming healthcare burden which is being increasingly recognized (Younossi et al., 2016; Tanajewski et al., 2017). For instance, a recent biomarker-based survey of population-wide cohorts revealed NAFLD prevalence in 21.9% of United States adults, with a quarter of these having fibrosis of stage F2 or higher (Wong et al., 2017). Notably, between 0.5 and 1.5% of adult individuals are likely to have hepatic fibrosis of stage F3 or higher (Wong et al., 2017) and are expected to develop a decompensated cirrhosis, on average, in 2.3 years or less (Hagstrom et al., 2017). In other developed countries, the prevalence of NAFLD is similar to that observed in the United States and Europe (Younossi et al., 2018), while its diagnoses in developing states are catching up proportionally to the growth of GDP (Zhu et al., 2015; Seyda Seydel et al., 2016). As an example, in Russia, a population-wide ultrasound screening has detected signs of NAFLD in 25–30% of individuals, while advanced fibrosis and cirrhosis were found in 2.3% and 0.8% cases, respectively (Drapkina et al., 2015). A similar prevalence of NAFLD (17–46%) and NASH (3–9%) were reported for European, American and Asian populations (Browning et al., 2004; Bedogni et al., 2005; Park et al., 2006; Zelber-Sagi et al., 2006; Lazo and Clark, 2008; Williams et al., 2011; Chalasani et al., 2012; Blachier et al., 2013; Fan, 2013; Li et al., 2014; Lazo et al., 2015; Younossi et al., 2018). Furthermore, NAFLD is frequently accompanied by other complications including cardiovascular diseases and type-2 diabetes, particularly in old-aged individuals (Leite et al., 2009; Chalasani et al., 2012; Schwenger and Allard, 2014). In fact, among the obese individuals and diabetic patients the incidence of NAFLD is between 70 and 90% (Targher et al., 2007, 2010).

The average prevalence of NAFLD in the developing countries has increased almost twofold over a decade (Fan, 2013), and has paralleled a rise in the incidence of NAFLD-associated disorders including metabolic syndrome, obesity, cardiovascular disease and type-2 diabetes (Lim et al., 2010). Interestingly, the growing incidence of both NAFLD and NAFLD-associated disorders strongly correlates with the dramatically increased *per capita* consumption of monosaccharide fructose (ingested mainly with sucrose) observed in the industrialized states within the last 30 years (Ouyang et al., 2008; Lim et al., 2010). Several case-controlled studies have further confirmed that the incidence of NAFLD correlates with the intake of either sugar-sweetened drinks (Assy et al., 2008; Abid et al., 2009) or fructose (Ouyang et al., 2008). The consumption of trans-unsaturated fats has been also attributed to higher NAFLD risks by some researchers (Tetri et al., 2008; Alkhouri et al., 2009). On the other hand, diets enriched with *cis*-unsaturated lipids have been shown to decrease intrahepatic fats accumulation and alleviate NAFLD (Nagao et al., 2005; Assy et al., 2009; Cussons et al., 2009). However, trans-unsaturated fats promote the formation of fatty liver only when accompanied by ingestion of carbohydrates (Lim et al., 2010). Consequently, long-term low carb high fat (LCHF) diets do not significantly increase the risk of NAFLD development (Stern et al., 2004; Lim et al., 2010). Overall, the elevating incidence of NALFD, especially in the well-developed countries, may stem from significantly increased daily caloric intake (Lim et al., 2010).

The observed trends and prevalence statistics prompt focusing on the non-alcoholic liver disease as a public health priority and necessitate implementing earlier detection of NAFLD in community-based healthcare settings. It is widely recognized that opportunistic screenings based on AST/ALT liver tests which are currently utilized by primary care physicians are insensitive and poorly specific for NAFLD (Drapkina et al., 2015; Petrick et al., 2015; Kwo et al., 2017).

## Current Techniques to Diagnose NAFLD and Their Limitations

Simple steatosis, which is the most common form of NAFLD, remains non-progressive in the majority of individuals and may resolve with proper modifications of lifestyle (Vilar-Gomez et al., 2015; Sung et al., 2016). Therefore, early population-wide diagnosis of NAFLD and, in particular, monitoring the transition of simple steatosis into NASH remains of paramount importance for the subsequent prevention of more severe and irreversible stages such as fibrosis and cirrhosis (Starley et al., 2010; Baffy et al., 2012; Michelotti et al., 2013). Currently, liver biopsy remains the only reliable option to determine NAFLD severity and to differentiate individuals with simple steatosis from NASH patients (Brunt, 2012). However, liver biopsy is (1) an invasive and expensive procedure; (2) associated with complications related to liver damage; (3) prone to sampling error and (4) limited in accessibility and reproducibility (Bedossa and Paradis, 2003; Nugent and Younossi, 2007; Sumida et al., 2014). Importantly, all three NAFLD-related pathophysiological processes (steatosis, inflammation of liver parenchyma and fibrosis) may co-exist within the same liver, and have a varied extent depending on particular regions; this variability is especially prominent when the extent of fibrosis is evaluated (Baranova et al., 2011). Along with the semi-quantitative nature of biopsies analysis (scoring), this fact can directly influence a reliability of the biopsy-based assessment of the liver status and complicate comparative studies of non-malignant conditions of human liver. The necessity for a non-invasive predictive staging of NAFLD also stems from the variability of its prognosis depending on an extent of the underlying histopathological changes in liver parenchyma.

Several non-invasive imaging strategies, including ultrasound, computer tomography (CT), magnetic resonance imaging (MRI) and proton magnetic spectroscopy, have been recently introduced to replace or complement biopsies; however, all these techniques are heavily dependent on skills of an operator and the availability of costly equipment. Less expensive types of imaging, including ultrasound, suffer from the lack of an objective quantitative analysis; while more robust and quantitative measurements such as proton density fat fraction (PDFF) scores (Noureddin et al., 2013) require specialized equipment that is rarely available at point-of-care stations (Di Martino et al., 2017). Finally, transient elastography, which is currently the most common non-invasive diagnostic modality for point-of-care assessment of NAFLD, is prone to significant sampling variability with probe location influencing diagnostic outcomes in at least 30% of patients (Zelber-Sagi et al., 2011).

Several approaches for NAFLD diagnostics are based on the detection of certain blood proteins and measuring individual clinical parameters. The predominant utility of these blood-based biomarkers is in the non-invasive estimation of liver fibrosis across the variety of conditions, including those related to chronic viral infections (Baranova and Younossi, 2008; Baranova et al., 2011). However, their sensitivity and specificity in the context of NAFLD remain highly limited (Baranova and Younossi, 2008). The commonly accepted NAFLD Fibrosis score (NFS), which is recommended by both the American Association for the Study of Liver Diseases (Chalasani et al., 2012) and the European Association for the Study of the Liver (European Association for Study of Liver and Asociacion Latinoamericana para el Estudio del Higado, 2015), differentiates a presence of the advanced fibrosis from mild or moderate fibrotic changes of stage 0–2 with an areas under the receiver operating characteristic curve (AUROC) in range of 0.82 – 0.85 (Angulo et al., 2007; Musso et al., 2011). When used as a sole screening tool, NFS places a considerable proportion of patients – between 20 and 58% – into indeterminate “gray zone” (Musso et al., 2011).

To some degree, “gray area” discrimination problems may be solved by combining several blood molecules and other clinical predictors into diagnostic or prognostic panels (Robin et al., 2009). Unfortunately, biomarker panels suffer from relatively low reproducibility of results when tested in independently collected sets of samples (Ein-Dor et al., 2006). One possible way to overcome these challenges is to develop biomarker panels for quantification of the particular pathophysiological process, rather than the overall “severity” of the condition which often reflects a variety of the histopathological changes confounding each other. Another way is to expand the search for reliable biomarkers into novel classes of the accessible molecules, for example, into circulating nucleic acids in general, and circulating miRNAs in particular.

**FIGURE 2:**
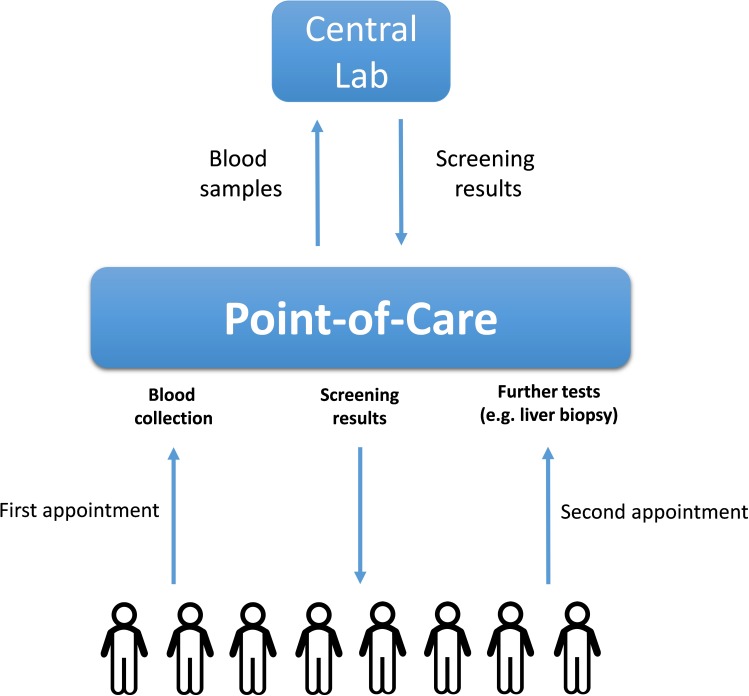
The logistics of a population-wide blood-based biomarkers examination. The blood samples collected initially at point-of-care stations upon first appointments with patients are transported to the central lab where they are analyzed for the presence of NAFLD-related soluble biomarkers (proteins or nucleic acids). After receiving the initial blood test results, the corresponding patients might be recommended to undergo further (more complex) tests including liver biopsies.

The ideal biomarkers for NAFLD would possess not only high specificity and sensitivity, but also be minimally invasive, inexpensive to measure and easily quantifiable. The biomarker utility of the molecules in the serum or the whole blood is well-recognized. Moreover, the collection of the venous blood is routinely performed at point-of-care stations, as it does not require the involvement of highly skilled personnel. As diagnostic or prognostic assessments of chronic liver disease do not require rapid turnaround time, the costs of blood biomarker assays may be driven down by shifting from point-of-care testing, which is usually more expensive (Lee-Lewandrowski and Lewandrowski, 2009), to a central laboratory. In turn, centralized diagnostic services are more suitable for implementing the tests which require highly standardized, specialized or moisture/temperature/human-error-sensitive laboratory equipment, while providing less of the challenges for maintaining regulatory compliance. The logistics described in **Figure 2** allows widening the spectrum of molecules suitable as biomarkers beyond commonly utilized serum proteins.

To conclude, significant limitations of the existing technologies pose a demand for highly sensitive, quantitative and non-invasive approaches suitable for routine diagnosis of NAFLD, as well as its stratification into risk groups and, finally, its response to lifestyle modifications and other treatments. Recently discovered circulating miRNA molecules offer great promises as a mine for novel biomarkers for a variety of human disorders. Importantly, miRNAs are renowned for their stability in the serum, making the separation of blood/serum collection and actual biomarker quantification steps feasible (**Figure 2**). Moreover, unlike proteins, nucleic acids can be detected by a polymerase chain reaction (PCR) that implies an exponential amplification of the original template; thus, circulating miRNAs might provide for much higher sensitivity as compared to protein biomarkers. The non-urgent character of NAFLD-related decision-making suggests NAFLD as an excellent test case for examining the practical utility of circulating miRNAs as biomarkers for longitudinal monitoring of human health. In addition, certain circulating miRNA profiles can reflect various histopathological events occurring in the liver and have a highly reliable predictive power to distinguished simple steatosis and NASH.

## Extracellular Circulating miRNA in the Diagnosis of Tissue Damage

MicroRNAs (miRNAs) are short (18–24 nt) non-coding RNA molecules that regulate gene expression by repressing the translation and enhancing the hydrolysis of target mRNAs (Ambros, 2004; Bartel, 2004). All miRNAs are originally transcribed as primary transcripts (pri-miRNAs), which are then hydrolyzed to shorter hairpin-carrying pre-mature miRNA (pre-miRNAs) molecules (Lee et al., 2003; Lee et al., 2004). The latter are exported into the cytoplasm where they are further cleaved to form mature 18–24 nt long single-stranded miRNAs (Liu et al., 2004; Meister et al., 2004; Chendrimada et al., 2005). Surprisingly, after the death of the parental cells, miRNAs remain stable in the nuclease-rich extracellular space for the prolonged periods predominantly due to their association with proteins of Argonaute family (Arroyo et al., 2011; Turchinovich et al., 2011; Turchinovich and Burwinkel, 2012). As a result, cell-free miRNAs have been consistently detected in all types of human biological fluids, including blood, urine, tears, breast milk, amniotic fluid, cerebrospinal fluid, saliva and semen (Turchinovich et al., 2012).

**FIGURE 3:**
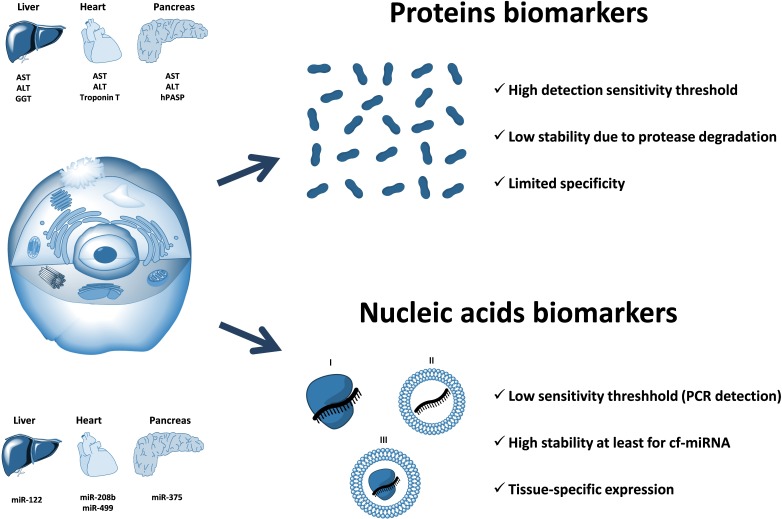
The advantage of circulating nucleic acids (including cell-free miRNA) over protein biomarkers. Circulating RNAs can be protected from nuclease degradation either by the associated proteins (I), encapsulation within the membrane vesicles (II), or both (III). Circulating nucleic acids-based biomarkers have a potential for a much higher sensitivity as compared to proteins due to the fundamentally more sensitive (PCR-based) methods of detection and quantification. In addition, circulating miRNAs are highly stable in extracellular fluids due to their association with proteins of Argonaute family and have a unique distribution of expression among different organs and tissues. Examples of organ-associated soluble protein biomarkers: ALT (alanine transaminase), AST (aspartate transaminase), GGT (gamma-glutamyltransferase), troponin T, hPASP (human pancreas-specific protein). Examples of organ-associated nucleic acids biomarkers: miR-122, miR-208b, miR-499, miR-375.

Multiple research reports have confirmed that cell-free circulating miRNA profiles reflect the well-being of the body and can be used to monitor the pathophysiological processes occurring in certain organs and tissues (Cortez et al., 2011; Etheridge et al., 2011; Sayed and Abdellatif, 2011). Furthermore, some circulating miRNAs, stabilized by Argonaute proteins and/or associated with membrane vesicles, are hypothesized to act as signaling molecules and mediate cell-to-cell communication between distant organs and tissues (Valadi et al., 2007; Hunter et al., 2008; Turchinovich et al., 2016). Finally, circulating miRNAs have been hypothesized to affect certain metabolic processes including those contributing to fatty live progression. Importantly, the techniques of proteins detection are fundamentally less sensitive than those developed for nucleic acids. Unlike proteins, nucleic acids (including miRNA) can be determined with an aid of a PCR that implies a multimillion amplification of the original template. Because of that, the detection of circulating miRNAs may be achieved with a much higher degree of sensitivity as compared to that for protein biomarkers (**Figure 3**). In theory, even a single miRNA molecule in the solution can be specifically detected by quantitative PCR.

The reports demonstrating a remarkable diagnostic capacity of circulating miRNAs began to appear shortly after their discovery in 2008. In a pioneering work, Wang et al. (2009) used acetaminophen-induced liver injury in the mouse model to document significant differences in the spectrum and levels of cell-free miRNAs in the blood of control and treated animals. In particular, serum levels of liver-specific miR-122 and miR-192 exhibited a dose-dependent increase that correlated with the rise of ALT activity; however, the changes of the miRNAs levels were detected significantly earlier (Wang et al., 2009). In another report, Laterza et al. (2009) have documented elevation of blood plasma concentrations of miR-122, miR-133a, and miR-124 that corresponded to injuries in liver, muscle and brain tissues, respectively. The activity of ALT and AST enzymes were both higher after the induction of cell death in either organ, while miR-122 and miR-133a increases were specific for the toxicity in liver and muscle, respectively. Furthermore, miR-122 exhibited a diagnostic sensitivity superior to that of ALT (Laterza et al., 2009). Likewise, the concentration of brain-specific miR-124 was significantly increased in blood plasmas of rats after the induction of brain ischemia, while the miR-122 and miR-133a levels remained at baseline values (Laterza et al., 2009).

In human studies, Zhang et al. (2010) have further confirmed that the change of miR-122 levels in the blood can be detected much earlier than the increase in liver aminotransferases activity. In addition, miR-122 changes were correlated with the histological status of the human liver and were specific for liver injury. Xu et al. (2011) have later shown that median serum levels of miR-21, miR-122, and miR-223 are significantly higher in chronic hepatitis and HCC patients as compared to that in healthy controls. Similar findings were also reported by an independent research group which proposed a microRNA panel, consisting of miR-122, miR-192, miR-21, miR-223, miR-26a, miR-27a, and miR-801, to diagnose HCC with high accuracy in chronic hepatitis B patients and individuals having liver cirrhosis (Zhou et al., 2011). This finding is essential, as it is more difficult to correctly evaluate the patients with underlying chronic pathologies for the presence of superimposing malignancy.

The diagnostic potential of cell-free miRNA to reflect the extent of cardiac muscle damage was also consistently reported. Thus, Corsten et al. (2010) have found that in AMI patients, the levels of cardiac myocyte-associated miR-208b and miR-499 were elevated 1600-fold and 100-fold, respectively as compared to that in healthy controls. Importantly, plasma miRNA levels were not affected by a wide range of clinical parameters, including age, gender, BMI, kidney function, systolic blood pressure, and white blood cell count (Corsten et al., 2010). Likewise, Akat and colleagues have documented up to 140-fold increases in blood levels of heart-specific circulating miRNAs during the onset of advanced heart failure, which coincided with a similar increase in cardiac troponin protein, the common marker for heart injury (Akat et al., 2014). Furthermore, the levels of those miRNAs decreased after the implantation of a ventricular assist device that amended myocardial cell death. The finding that a cardiac damage initiates the detectable release of cardiomyocyte-specific miRNAs into the circulation gives promise for an early detection of impending myocardial infarction.

The miRNA-375, which is expressed at high levels only in the pancreas cells, has shown a great potential as a biomarker for β-cells death and an early predictor of diabetes. In mice, administration of high-doses of streptozotocin led to substantial increases in the blood levels of cell-free miR-375, detected prior to the onset of hyperglycemia (Erener et al., 2013). In addition, in mouse models of autoimmune diabetes, circulating miR-375 levels were dramatically higher 2 weeks before the onset of the diabetes (Erener et al., 2013). In another report, Higuchi et al have documented a remarkable increase in miR-375, as well as miR-101 and miR-802 content, in the sera of type-2 diabetes patients as compared to subjects with unimpaired glucose tolerance (Higuchi et al., 2015).

Similar to all other tissues, malignant tumors also release their endogenously expressed miRNAs into the extracellular environment. Indeed, many cancer-specific miRNAs have been consistently found in patients’ blood plasma/serum at different stages of the disease (Mitchell et al., 2008; Skog et al., 2008; Taylor and Gercel-Taylor, 2008; Rabinowits et al., 2009). Besides tumor-derived miRNAs, the diagnostic relevance of circulating miRNAs originating from cells within the tumor microenvironment (including the immune cells) has been also extensively validated (Schwarzenbach et al., 2014).

Importantly, most (if not all) research works were so far focused on liver-, muscle-, cardiac-, pancreas-, immune cells- and tumor-specific miRNAs. The recently generated human miRNA expression atlas contains the data on the abundance of 1997 miRNAs in 61 biopsies from different organs (Ludwig et al., 2016). While most miRNAs (82.9%) are not specific for a single tissue, many distinct miRNAs and miRNA families are predominantly expressed in certain cell types (Ludwig et al., 2016). Interestingly, for many organs, inter-organism variability of organ-specific miRNA expression was significantly lower than their inter-organ variability (Ludwig et al., 2016).

Thorough description of each reported miRNA signature associated with certain diseased states goes well beyond the scope of this review. Despite great strides toward clinical acceptance, the diagnostic utility of circulating miRNAs for multiple pathologies remains to be tested and validated. In the following part of the article, we will concentrate on the utility of circulating miRNA for diagnosis and risk stratification of NAFLD.

## Extracellular Circulating miRNA Profiles for the Diagnosis of NAFLD and Associated Disorders

Multiple previous studies have been focused on discriminating various NAFLD stages by analyzing miRNA expression profiles in liver parenchyma (Guo et al., 2016; Soronen et al., 2016; Latorre et al., 2017). However, only several research groups have so far investigated NAFLD-associated changes in the spectrum of extracellular miRNAs present in human blood (Cermelli et al., 2011; Yamada et al., 2013; Miyaaki et al., 2014; Tan et al., 2014; Becker et al., 2015; Pirola et al., 2015; Akuta et al., 2016; Liu et al., 2016; Raitoharju et al., 2016; Salvoza et al., 2016; Thompson et al., 2017) (**Table 1**). These reports strongly suggest that circulating miRNAs can be utilized as more sensitive and specific biomarkers of liver damage as compared to currently employed biochemical tests based on measuring the relative activity of serum transaminases.

**Table 1:** Current reports of circulating miRNA expression in NAFLD individuals.

Upregulated cf-miRNAs	Number of participants	Reference
miR-122, miR-34a, miR-16	19 healthy vs. 34 NAFLD	Cermelli et al., 2011
miR-122, miR-21, miR-34a, miR-122, miR-451	311 healthy vs. 92 NAFLD	Yamada et al., 2013
miR-122	17 mild NAFLD vs. 34 severe NAFLD	Miyaaki et al., 2014
miR-122; miR-1290, miR-27b, miR-192	90 healthy vs. 152 NAFLD	Tan et al., 2014
	80 healthy vs. 103 NAFLD	
miR-122, miR-192, miR-19a, miR-19b, miR-125b, miR-375	16 healthy vs. 16 simple steatosis vs. 16 NASH	Pirola et al., 2015
	19 healthy vs. 30 simple steatosis vs. 47 NASH	
miR-122, miR-21, miR-192	61 healthy vs. 137 NAFLD	Becker et al., 2015
miR-122, miR-34a	36 healthy vs. 28 NAFLD	Salvoza et al., 2016
miR-122, miR-885	724 healthy vs. 147 NAFLD	Raitoharju et al., 2016
miR-122	36 NAFLD patients (different stages)	Akuta et al., 2016
miR-122, miR-122b, miR-146b, miR-16, miR-192, miR-21, miR-27b, miR-34a	37 healthy vs. 48 NAFLD (17 simple steatosis, 31 NASH)	Liu et al., 2016
16 miRNAs, with largest increases detected in levels of miR-122 and miR-199a	20 obese children with NAFLD and 10 healthy controls	Thompson et al., 2017


In a pioneering work, Cermelli et al. (2011) observed that serum levels of miR-122, miR-34a and miR-16 were substantially higher in NAFLD patients than in healthy controls and were positively correlated with the disease stage. On average, in NAFLD patients the levels of miR-122 and miR-16 were increased by 7.2-fold and 5.5-fold, respectively, as compared to controls, while miR-34a content had risen from undetectable levels to about of 10000 copies per mL of serum. Furthermore, patients with simple steatosis exhibited 5.7-fold and 5.3-fold increases in levels of miR-122 and miR-16, respectively, while in the NASH group these levels were further up to 2–3-fold higher as compared to those in the simple steatosis cohort. Importantly, steady increases of concentrations of the same miRNAs were also observed along the course of acute HCV infection (Cermelli et al., 2011). While serum levels of miR-122 and miR-16 were correlated with ALT and AST enzyme activities, on receiver–operator characteristic (ROC) curve analysis these miRNAs performed significantly better than the enzymes. Later, gradual increases in serum levels of miR-122 in parallel with the progression of NAFLD were independently reported by several other research groups (Yamada et al., 2013; Miyaaki et al., 2014; Pirola et al., 2015).

In particular, Yamada et al. (2013) assessed the presence of intrahepatic steatosis and the blood serum levels of five selected miRNAs (miR-21, miR-34a, miR-122, miR-145, and miR-451) in 403 Japanese subjects who attended ordinary health examinations. The serum level of four out of five miRNAs (miR-21, miR-34a, miR-122, and miR-451) were markedly higher in participants with NAFLD (92 out of 403), moreover, the levels of miR-122 were correlated with the severity of steatosis. The study of Miyaaki and colleagues, which was also performed on Japanese patients, has further confirmed a strong correlation of miR-122 expression in serum with the severity of steatosis (Miyaaki et al., 2014). Interestingly, serum miR-122 levels correlated to the liver fibrosis stage inversely, with lower miR-122 expression levels associated with the advanced fibrosis (Miyaaki et al., 2014). This observation agrees with the fact that hepatocytes are the main source of miR-122 in the blood (Chang et al., 2004). Indeed, liver fibrosis is accompanied by a persistent replacement of miRNA-producing hepatic cells with the extracellular matrix.

In another, more technically advanced study, circulating miRNA panel, consisting of miR-122, miR-1290, miR-27b, and miR-192 showed its high diagnostic accuracy for NAFLD (Tan et al., 2014). The expression patterns within total circulating miRNAs were initially detected by deep sequencing of RNA isolated from 20 controls and 20 NAFLD sera. Subsequently, real-time PCR assay was applied to measure the levels of selected miRNAs in training (90 healthy vs. 152 NAFLD patients) and validation (80 healthy vs. 103 NAFLD patients) cohorts of participants. The resultant miRNA panel showed sensitivity and specificity superior to that of ALT and FIB-4 tests and had satisfactory diagnostic performance regardless of the NAFLD activity score (NAS) status (Tan et al., 2014).

By using global serum profiling of 84 different miRNAs, Pirola et al. (2015) identified six circulating miRNA species which were upregulated more than 2-fold in individuals with either simple steatosis or NASH. The most dramatic fold changes were observed in levels of miR-122 which showed 7.2-fold higher expression in the sera of NASH patients vs. controls, and 3.1-fold change in NASH patients vs. individuals with simple steatosis (Pirola et al., 2015). Subsequent ROC analysis revealed that three miRNAs (miR-122, miR-192, and miR-375) could differentiate NASH and simple steatosis, however, only miR-122 levels were instrumental in distinguishing liver fibrosis. It has to be mentioned that another research group was not able to correlate serum levels of miR-122 to histological features of NAFLD in the presence of inflammation, while still confirming the elevation of its concentrations in the blood of NAFLD patients well over the baseline (Salvoza et al., 2016). Finally, one research study has failed to detect the differences in miR-122 levels between NAFLD and controls (Celikbilek et al., 2014).

Becker et al. (2015) showed that serum levels of miR-122, miR-192, and miR-21 strongly correlate with the levels of various known NASH biomarkers and isolated pathophysiological parameters reflecting NASH severity, including a degree of steatosis, ballooning, lobular inflammation and fibrosis. Subsequently, serum concentrations of these miRNAs were used as inputs into a relatively simple scoring system with a range from 0 to 3, which could be used for a non-invasive prediction of NASH either alone, or in combination with similarly scored levels of cytokeratin-18 fragment Asp396 (CK18-Asp396) that was previously described as a biomarker of NAFLD progression (Younossi et al., 2008). Surprisingly, the combined miRNA scoring model had the same diagnostic performance for discriminating NASH as CK18-Asp396 fragment serum levels, while adding the CK18-Asp396 to the three-miRNA profiles improved the diagnostic efficiency of the resultant panel only marginally (from AUCOR of 0.81 to AUROC of 0.83) (Becker et al., 2015).

Raitoharju et al. (2016) reported the association of blood miR-122 and miR-885 concentrations with ultrasonically assessed fatty liver in Finns study participants aged 34–49 years. As this study cohort was unusually large, the confidence of the reported findings was impressive; thus, Bonferroni-corrected *p*-values for miR-122 and miR-885 constituted 1.92 × 10^-15^ and 2.58 × 10^-4^, respectively. For the prediction of NAFLD, the levels of miR-122 were comparable in its performance to liver transaminases levels. However, combining miR-122 and miR-885 levels with common NAFLD risk factors improved the risk stratification only marginally; therefore, the authors were convinced against the clinical value of blood miRNA levels for the diagnostics of NAFLD in general population (Raitoharju et al., 2016).

In the research reports discussed above (**Table 1**), only circulating miR-122 was consistently shown to be upregulated in sera of NAFLD individuals. Moreover, its serum levels were affected by significantly higher degree as compared to other miRNAs. These observations also accord well with the fact that miR-122 is expressed almost exclusively in the hepatocytes, comprising up to 70% of the total pool of liver miRNAs (Chang et al., 2004). Besides miR-122, several inflammation-related circulating miRNAs including miR-21, miR-34a, miR-451, miR-200a, miR-199, and miR-155 could be strongly associated with NAFLD and contribute to liver inflammation, fibrosis, and cirrhosis (Cheung et al., 2008; Murakami et al., 2011; Sun et al., 2014; Ding et al., 2015). Therefore, the elevation of these miRNAs’ levels in the blood circulation could be anticipated.

Finally, NAFLD is frequently accompanied by the associated disorders such as type-2 diabetes and various cardiovascular pathologies. While the precise mechanistic links between NAFLD and those diseases are not completely understood, monitoring of extracellular miRNAs derived from non-liver tissues commonly affected in NAFLD patients may be of value. In particular, the detection of pancreas-derived miR-375 and cardiac muscle-specific miR-208b/miR-499 in human biological fluids could further enhance the sensitivity and specificity of diagnosis of NAFLD severity (Corsten et al., 2010; Erener et al., 2013; Akat et al., 2014; Higuchi et al., 2015).

The description of putative biological roles of miR-122 and other NAFLD-associated circulating miRNAs goes well beyond the scope of this manuscript. However, those miRNAs may contribute to various pathological processes by targeting certain genes involved in key intracellular pathways. For instance, miR-122 has been shown to regulate proliferation, apoptosis and epithelial-to-mesenchymal transduction (EMT) in HCC cells by targeting the Wnt/β-catenin pathway (Xu et al., 2012; Jin et al., 2017). In addition, Wnt/β-catenin signaling directly controls the expression of the laminin-5 gene whose product enhances the invasiveness of cancer cells by favoring cell-substrate adhesion (Hlubek et al., 2004). Therefore, downregulation of miR-122 could facilitate the formation of tumor promoting microenvironments in the liver by inducing EMT and remodeling the laminin-containing extracellular matrix. Indeed, miR-122 was significantly downregulated in liver cancers with intrahepatic metastasis, while the restoration of miR-122 expression in metastatic HCC cells in culture decreased their motility and invasiveness (Tsai et al., 2009).

The initial appeal of circulating miRNA molecules for the diagnosis of various liver diseases has not so far resulted in a substantial improvement over the existing (imperfect but inexpensive) techniques. It is likely that different miRNAs play distinct roles at different stages of NAFLD development, or that more intricate interplay between miRNAs, soluble proteins and various NAFLD-associated pathophysiological processes is in place (Makarova et al., 2016; Galatenko et al., 2018). Therefore, from diagnostic and stratification standpoints, circulating miRNAs should be combined into a diagnostic panel based on their ability to reflect individual pathophysiological components, which intertwine in NAFLD, and define the course and the outcome of the disease in a given individual. Moreover, concentrations of individual miRNAs in the serum may not be collinear with the temporal pattern of deterioration of liver parenchyma.

## Current Limitations and Emerging Technologies of cf-RNA Detection

The molecular methods employed so far for the detection and characterization of extracellular nucleic acids include microarrays, RT-qPCR and next-generation sequencing (NGS) (Cortez et al., 2011; Etheridge et al., 2011). Microarrays had been widely used to define circulating miRNA expression in early reports. However, due to their limited sensitivity microarrays can only screen the most abundant miRNAs in biofluids. On the contrary, both RT-qPCR and NGS can detect low abundant miRNAs and remain currently the methods of choice. Even though NGS allows both the discovery of novel miRNAs and the identification of other RNA species, until recently it was associated with significant costs, labor-intensiveness and requirement of high RNA inputs which are hardly obtainable from adequate sample volumes of liquid biopsies. In contrast, RT-qPCR is a more convenient, sensitive and cost-effective approach, however, its ability to detect other RNAs (including fragments derived from mRNAs and lncRNAs), which are also present in the extracellular fluids (Turchinovich et al., 2014; Freedman et al., 2016; Wei et al., 2016; Yuan et al., 2016; Yeri et al., 2017; Max et al., 2018), is limited due to their highly degraded state. Another disadvantage of RT-qPCR-based methods is a relatively high false-positive rate that could partially explain multiple inconsistent results among various studies on circulating miRNA as biomarkers (Turchinovich et al., 2016).

A number of other factors can affect circulating RNA quantification and may accord for the inconsistency among independent research reports. For instance, a certain level of hemolysis that occurs during the collection of blood samples can mask those circulating miRNAs which are also present in blood cells. Thus, human erythrocytes contain high levels of miR-451a, miR-16 and miR-21 and, therefore, their cell-free levels in blood plasma/serum can be significantly altered upon hemolysis. Likewise, the lysis of other blood cell types can affect extracellular levels of certain miRNAs by up to 50-fold (Pritchard et al., 2012). Therefore, some previously reported data (including diagnostic miRNA panels) may require re-evaluation and validation on the adequately processed blood samples. Finally, the heterogeneity of technical methods employed for the detection and characterization of circulating miRNA signatures in the past imposes a more comprehensive standardization and harmonization of biological assays.

Until recently, cell-free RNAs in biological fluids have been analyzed almost exclusively with qPCR-based methods and were limited to only miRNA detection. The advent of NGS has enabled detection of the whole spectrum of RNA species in extracellular fluids; nonetheless, sequencing of trace amounts of RNA remained a major challenge (Turchinovich et al., 2014; Freedman et al., 2016; Wei et al., 2016; Yuan et al., 2016; Yeri et al., 2017; Max et al., 2018). Most currently available commercial kits and published protocols for NGS library preparation rely on significantly higher inputs of RNA than those obtainable from standard volumes of liquid biopsy samples. However, a number of highly sensitive NGS library preparation approaches has been suggested and are available on the market since recently. For instance, Capture and Amplification by Tailing and Switching (CATS) technology allows deep sequencing of low amounts of nucleic acids with unprecedented sensitivity (Turchinovich et al., 2014). Unlike other methods, CATS can generate NGS libraries from pictogram inputs of highly fragmented nucleic acids and allows characterization of the whole spectrum of circulating RNAs in biological fluids including mRNA and lncRNA (Turchinovich et al., 2014). Importantly, while the capacity of cell-free miRNAs for diagnosis of various disorders has been assessed in many studies, the relevance of other circulating RNA species has not been addressed so far.

## Conclusion and Future Perspectives

Despite multiple research reports demonstrating amazing promises of circulating miRNAs for diagnostic application, this field is still in its infancy. Primarily, harnessing novel more sensitive nucleic acids detection technologies is necessary to confirm whether cell-free miRNAs can reflect the physio-pathological status of the liver in NAFLD patients and to serve as early-stage fingerprints of the fatty liver in biological fluids. Secondly, ultrasensitive NGS-based approaches have not yet been applied to characterize the changes in overall cell-free RNAs occurring in the biofluids of NAFLD individuals. It remains to be tested whether other RNA species (including mRNA and lncRNA), which are also highly abundant in the blood plasma, could serve as more reliable biomarkers for NAFLD condition as compared to common liver enzymes tests. Finally, determining correlations of extracellular RNAs changes with an early onset of NAFLD-related conditions remains of paramount interest. Developing a simple, highly reliable, cost-efficient and non-invasive diagnostic test system to screen and identify early NAFLD stages without the use of a liver biopsy would significantly reduce both the mortality and the economic burden associated with NAFLD and related diseases.

Importantly, liver biopsy, along with other approaches which are highly dependent on skilles of an operator and the availability of costly equipment could hardly fit into a model of screening tests performed in point-of-care stations. Therefore, despite inherent limitations of blood biomarkers (Ein-Dor et al., 2005, 2006; Veytsman and Baranova, 2015), the absence of clear alternatives prompts the development of functionalized diagnostic panels where each individual component would, ideally, reflect certain pathophysiological process contributing to the NAFLD progression in a given individual and predict its outcome. The fundamental advantage of circulating RNA over protein biomarkers is that, unlike proteins, nucleic acids can be detected by a PCR which has, in theory, the sensitivity threshold of a single molecule. Finally, miRNA-based diagnostic panels could, if necessary, be augmented by other blood-based biomarkers including liver-specific proteins or metabolites.

## Author Contributions

ATo and ATu conceived the study and coordinated the work. ATu and AB prepared the tables and figures and wrote the manuscript. ATu, AB, ATo, and OD participated in manuscript design. AB and OD provided expert’s opinion on the content and critical revision.

## Conflict of Interest Statement

The authors declare that the research was conducted in the absence of any commercial or financial relationships that could be construed as a potential conflict of interest.
